# A new gene tree algorithm employing DNA sequences of bovine genome using discrete Fourier transformation

**DOI:** 10.1371/journal.pone.0277480

**Published:** 2023-03-09

**Authors:** Roxana Abadeh, Mehdi Aminafshar, Mostafa Ghaderi-Zefrehei, Mohammad Chamani

**Affiliations:** 1 Department of Animal Science, Science and Research Branch, Islamic Azad University, Tehran, Iran; 2 Department of Animal Science, Yasouj University, Yasouj, Iran; University of Helsinki, FINLAND

## Abstract

Within the realms of human thoughts on nature, Fourier analysis is considered as one of the greatest ideas currently put forwarded. The Fourier transform shows that any periodic function can be rewritten as the sum of sinusoidal functions. Having a Fourier transform view on real-world problems like the DNA sequence of genes, would make things intuitively simple to understand in comparison with their initial formal domain view. In this study we used discrete Fourier transform (DFT) on DNA sequences of a set of genes in the bovine genome known to govern milk production, in order to develop a new gene clustering algorithm. The implementation of this algorithm is very user-friendly and requires only simple routine mathematical operations. By transforming the configuration of gene sequences into frequency domain, we sought to elucidate important features and reveal hidden gene properties. This is biologically appealing since no information is lost via this transformation and we are therefore not reducing the number of degrees of freedom. The results from different clustering methods were integrated using evidence accumulation algorithms to provide in insilico validation of our results. We propose using candidate gene sequences accompanied by other genes of biologically unknown function. These will then be assigned some degree of relevant annotation by using our proposed algorithm. Current knowledge in biological gene clustering investigation is also lacking, and so DFT-based methods will help shine a light on use of these algorithms for biological insight.

## Introduction

Periodic patterns can be tracked across biological sequences. By locating and characterizing DNA patterns, some biologically motivated information can be extracted about the structure and function of the DNA sequence [1, 2]. To analyze DNA sequence it should first be converted into numerical sequence. Different mapping functions have been defined for this purpose [3].The simplest one is binary mapping that should bear up biological implication like electron-ion-interaction pseudo-potential (EIIP) mapping rule [4]. A new binary mapping function based on weak-strong hydrogen bonding has also been defined [5]. The paper by Arniker and Kwan (2012) thoroughly covers most of the main mapping functions. From a digital signal processing (DSP) point of view, a DNA sequence is a one-dimensional signal and it is therefore possible to apply many signal processing algorithms to explore DNA sequence data [6]. When a new DNA sequence with unknown function is identified, generally researchers quest for the most ’similar’ or ’identical’ DNA sequence in a well-annotated biological dataset. This is the accepted routine, since identical DNA sequences tend to reflect identical biological functionality and forms the basis for determining whether the DNA sequences are homologous, e.g., there is shared ancestry between them. DSP based methods have been used to locate reading frames in DNA, including different gene regions (exons) [7], to detect splice sites within the gene [8], to identify active sites in a protein, to identify acceptor splicing sites and motif patterns in DNA [9, 10]. DFT is the most popular DSP technique [7]. In general, the fast Fourier transform was developed to compute the DFT. In fact, DFT has been used to analyze DNA sequence data [8]. Discrete Fourier Transform (DFT) is a powerful tool in many practical applications, such as analysis of biological events. For example, DFT has been used to develop a measure of DNA sequence similarity [11], to quantify the physical behavior of biological systems [12], as well as to cluster and predict gene expression [13–15]. Recently, Hoang et al. (2015) proposed a new Fourier power spectrum based method to cluster DNA sequences in which sequences of different length could be easily compared. The spectrum estimation is mainly performed in random processes in which random variables cannot be thoroughly explored by means of deterministic tools. In this study we used DFT to cluster the major bovine milk genes. The nature of this method can be applied to data consisting of any sized DNA. However, Fourier analysis that is well-adapted to the study of biological data can be used instead of conventional modeling techniques. It would seem that its application and utility shall increase as more and more high-throughput data become available.

### A simple illustration of DFT of DNA sequence

The Fourier transform (FT) of a continuous-time function x(t) can be shown as follows [8]:

$$X(f)=F\{x(t)\}=\int_{-\infty }^{+\infty }{x(t)e}^{-j2\mathrm{\pi ft}}\;dt$$

Where $j=\sqrt{-1}$. Since the Fourier transform is an invertible function, the inverse of FT is given by:

$$x(t)=F^{-1}\{X(f)\}=\frac{1}{2\pi }\int_{-\infty }^{+\infty }{X(f)e}^{-j2\mathrm{\pi ft}}\;df$$

The Discrete Fourier Transform (DFT) of x[n] with period *N* at the point of *k* is defined as below [8]:

$$X[k]=F\{x[n]\}=\sum_{n=0}^{N-1}x[n]e^{-j2\pi kn/N}$$

Also, the inverse of DFT is given by:

$$x[n]=F^{-1}\{X[k]\}=\frac{1}{N}\sum_{n=0}^{N-1}X[k]e^{j2\pi kn/N}$$

Fundamental to both continuous and discrete Fourier transform are the following equations:

$$\sin\left(nx\right)=\frac{e^{jnx}-e^{-jnx}}{2j},\;\cos\left(nx\right)=\frac{e^{jnx}+e^{-jnx}}{2}$$

Now suppose we have the following gene: *G* = {a,g,c,t,t,t,a}. This postulated gene, has 7 nucleotides (bases). Here, the conversion of DNA sequence to a numerical sequence is necessary. We can define an arbitrary and randomly value-assignment scheme such as: 5 to A, 6 to C, 7 to G and 8 to T. However, it would be better to write down a value-assignment scheme that converts a DNA sequence into four binary sequences. This scheme, we call it *G*−*scheme*, generates *x*[*n*] sequence in which *i*−*th* item of this sequence, *x*_*i*_[*n*], can be computed by:

$$x_{i}[n]=\left\{\begin{array}{c} 1\;ifG[j]=j_{i}\\ 0\;otherwise \end{array}\right\}(j\in \{A,C,G,T\})$$

Therefore, for the above example, we consider that *N* = 7, then, we could have the following binary indicator sequences:

w[n]=G[a]={1,0,0,0,0,0,1}

x[n]=G[t]={0,0,0,1,1,1,0}

y[n]=G[g]={0,1,0,0,0,0,0}

z[n]=G[c]={0,0,1,0,0,0,0}

By computing the DFT of the *w*_*n*_, *W*[*k*] = F{*w*[*n*]}, for *k* = 0 *to* 7,

$$W[k]=\sum_{n=0}^{N-1}w[n]e^{-j\frac{2\pi kn}{N}}$$

we have:

W[k]={2,1.623+0.782j,0.777+0.975j,0.099+0.434j,0.099‐0.434j,0.777‐0.975j,1.623‐0.782j}.

In addition, we can find the DFT of the *x*_*n*_, *y*_*n*_ and *z*_*n*_ as follows:

X[k]={3,‐2.024+0.975j,0.346‐0.434j,0.178‐0.782j,0.178+0.782j,0.346+0.434j,‐2.024‐0.975j},

Y[k]={1,0.623‐0.782j,‐0.223‐0.975j,‐0.901‐0.434j,‐0.901+0.434j,‐0.223+0.975j,0.623+0.782j},

Z[k]={1,‐0.223‐0.975j,‐0.901+0.434j,0.623+0.782j,0.623‐0.782j,‐0.901‐0.434j,‐0.223+0.975j}, respectively.

For each DNA bases, we can write down a spectrum and DFT can be applied. In the above, just *G[a] = {1*, *0*, *0*, *1}* is shown as DFT. In this study, we use DFT to handle the DNA sequences of a set of candidate genes governing milk production in cattle. To the best of our knowledge, there have been no previous studies in this area.

## Materials and methods

### Data extraction and conversion

In the study, we randomly selected 30 genes involved in bovine milk production [16]. The DNA sequences of these genes were obtained from the NCBI genome database (http://www.ncbi.nlm.nih.gov/genbank/gene) and downloaded as FASTA format. To extract all gene features (e.g. exon number, exon length, gen length, etc) automatically, we developed a stand-alone program using C Sharp programming language. This simple program facilitated retrieval of gene features from stored DNA sequence data. This software is available from authors upon request. In this study 30 genes that are functioning in milk production (based upon 6 classifications), were randomly selected.

### Gene clustering by means of DFT

After converting gene sequences into respective binary indicator sequences using binary mapping, they were discrete Fourier transformed. In this way, we used the idea that was proposed by [17]. Simply, we first tried to find DFT of an indicator binary sequence at frequency k (k = 0, …, N−1) and later on, the power spectrum of that indicator sequence at frequency k was obtained. For comparing a set of numerical sequences, those sequences should be of the same length in order to for example a metric measure like Euclidian distance could be applicable to find their distance with each other. As Table 1 in this paper shows, the length of applied genes, therefore, their respective binary indicator sequences were quite different. To tackle with this issue, we used new mathematical moments e.g. Power Spectrum Moments (PSM) method by [17]. The result obtained by PSM was used as an entry into four main agglomerative hierarchical clustering algorithms e.g. nearest distance (single linkage method), furthest distance (complete linkage method), un-weighted pair group method average (UPGMA, group average), weighted pair group method centroid (WPGMC) and gene trees were obtained. In order to combine the results of these four gene trees into single one (sort of ensemble clustering), we applied a so called evidence accumulation [18]. This was done to tackle with sensitivity with distance measure as it was hard to find the proper one, possible noise and outlier and cluster type, shape and density of single algorithm. In this way, we converted clustering results of an algorithm into binary distance matrices and evidence accumulation was obtained from collective of the binary distance matrices and finally a hierarchical clustering algorithm was used to learn the ensemble-like cluster. At the end, the GeneMANIA prediction tool was used to check the evidence accumulation results with regard biological function [19]. To do so, a gene list of every singleton of final ensemble cluster was obtained and imported to GeneMANIA. This program tries to find those genes that are functionally similar, or have shared properties with the list of imported genes, and displays an interactive functional association network at seven levels: predicted, physical interactions, co-expression, co-localization, pathway, shared protein domains and genetic interactions. All computations were performed using MATLAB, Version 2015.

**Table 1:** The features of 30 randomly selected genes involved in bovine milk production.

No	Gene symbol	Gene Id	Length	Exon count	Chr	mRNA	Protein	Nucleotide	Minus strand	Total annotated spliced exon length	A	C	G	T	N	GC Content (%)
**1**	EZR	281574	45027	13	9	NM_174217	NP_776642.1	NC_007307.5	from: 99294295 to: 99339321	2704	10562	10497	11919	12049	-	49.8
**2**	CSNK1A1	282684	47003	10	7	NM_174711	NP_777136.1	NC_007305.5	from: 61313755 to: 61266753	1436	13741	8407	9285	15319	251	37.6
**3**	ACTR3	281597	46960	11	2	NM_174226	NP_776651.1	NC_007300.5	from: 68049206 to: 68096165	2449	13365	7763	8697	16768	367	35
**4**	ENO1	281141	13678	12	16	NM_174049	NP_776474.2	NC_007314.4	from: 41629401 to: 41643078	1790	3055	3450	3570	3553	50	51.3
**5**	DHRS3	281482	49779	7	16	NM_174180	NP_776605.2	NC_007314.4	from: 37825242 to: 37875020	1908	12284	12354	12742	12204	195	50.4
**6**	RAB2A	508373	45028	6	14	NM_001075354	NP_001068822.1	NC_007312.5	from: 26027651 to: 26072678	1638	12951	8578	8982	14467	50	39
**7**	VDAC1	282119	25717	13	7	NM_174485	NP_776910.2	NC_007305.5	from: 44854066 to: 44828350	1783	6099	5491	6415	7712	-	46.3
**8**	VIM	280955	7930	9	13	NM_173969	NP_776394.2	NC_007311.5	from: 31432088 to: 31440017	1849	2178	1751	1707	2294	-	43.6
**9**	LCP1	540990	64463	16	12	NM_001034720	NP_001029892.1	NC_007310.5	from: 15287343 to: 15222881	2103	17325	14116	13243	19779	-	42.4
**10**	FGG	280792	7472	9	17	NM_173911	NP_776336.1	NC_007315.5	from: 3080658 to: 3088129	1611	2262	1375	1476	2359	-	38.1
**11**	HSPA1A	282254	2101	1	23	NM_203322	NP_976067.2	NC_007324.5	from: 26305063 to: 26302963	2101	472	608	723	298	-	63.3
**12**	STX3	513275	79347	11	15	NM_001101971	NP_001095441.1	NC_007313.5	from: 83698466 to: 83777812	1349	19805	18180	18788	22074	500	46.6
**13**	ABCG2	536203	70516	16	6	NM_001037478	NP_001032555.2	NC_007304.5	from: 37260866 to: 37331381	2186	19550	13403	14323	23078	162	39.3
**14**	CD14	281048	1417	2	7	NM_174008	NP_776433.1	NC_007305.5	from: 51037894 to: 51036478	1327	280	443	422	272	-	61
**15**	ARHGDIA	338054	4016	6	19	NM_176650	NP_788823.1	NC_007317.5	from: 52103204 to: 52107219	1852	664	1203	1325	824	-	62.9
**16**	MUC1	281333	4098	9	3	NM_174115	NP_776540.1	NC_007301.5	from: 16573975 to: 16578072	2052	886	1316	1083	813	-	58.5
**17**	CD9	280746	34828	8	5	NM_173900	NP_776325.1	NC_007303.5	from: 110441683 to: 110406856	1199	7734	8795	8757	8816	726	50.4
**18**	TMBIM1	404134	17550	12	2	NM_205798	NP_991367.1	NC_007300.5	from: 112246156 to: 112228607	2206	3492	4700	5007	4351	-	55.3
**19**	VAT1	510698	6811	6	19	BC148900	NP_001179194.1	NC_007317.5	from: 44069099 to: 44062289	2725	1327	1832	1920	1657	75	55.1
**20**	KRT6A	614456	5254	9	5	NM_001083510	NP_001076979.1	NC_007303.5	from: 30244905 to: 30250158	2166	1423	1213	1351	1267	-	48.8
**21**	ARF1	338058	18063	5	7	NM_176653	NP_788826.1	NC_007305.5	from: 2947622 to: 2929560	1797	4185	3962	4865	5051	-	48.7
**22**	CFL1	534553	3196	4	29	NM_001015655	NP_001015655.1	NC_007330.5	from: 45680608 to: 45677413	883	554	937	1018	687	-	61.2
**23**	GNB1	281201	77959	11	16	NM_175777	NP_786971.2	NC_007314.4	from: 48085918 to: 48163876	2940	19075	16646	19000	22916	322	45.7
**24**	HSPA5	415113	4160	9	11	NM_001075148	NP_001068616.1	NC_007309.5	from: 99410000 to: 99405841	1929	969	664	819	935	773	35.6
**25**	CORO1A	282196	5415	11	25	NM_174521	NP_776946.1	NC_007326.5	from: 28003778 to: 27998364	1575	982	1611	1811	1011	-	63.2
**26**	YWHAG	286862	1152	1	25	NM_174793	NP_777218.2	NC_007326.5	from: 36521246 to: 36520095	1152	304	273	302	273	-	49.9
**27**	P4HB	281373	9945	12	19	NM_174135	NP_776560.1	NC_007317.5	from: 52113320 to: 52123264	2317	2087	2507	2902	2449	-	54.4
**28**	SAR1A	517171	11301	7	28	NM_001034521	NP_001029693.1	NC_007329.5	from: 25557334 to: 25546034	758	2985	2156	2328	3832	-	39.7
**29**	HSP90AA1	281832	5368	11	21	NM_001012670	NP_001012688.1	NC_007319.5	from: 67017468 to: 67012101	3041	1405	1068	1445	1450	-	46.8
**30**	RAB1A	539339	28842	5	11	NM_001033628	NP_001028800.1	NC_007309.5	from: 65246768 to: 65217927	1328	8440	4717	5790	9895	-	36.4

## Results and discussion

**Fig 1:**
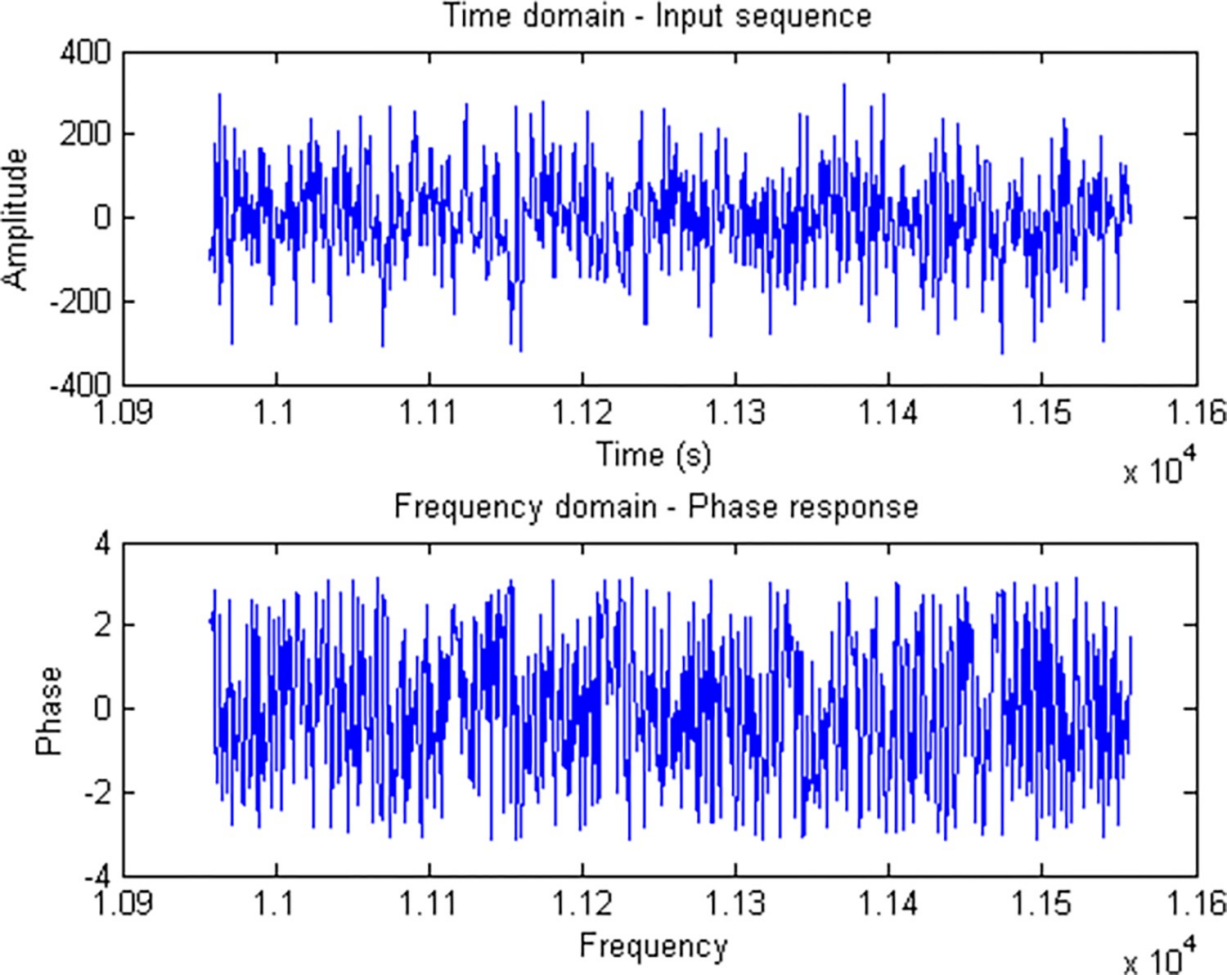
Comparison of sequence similarity from Fourier frequency domain for all 30 genes.

**Fig 2:**
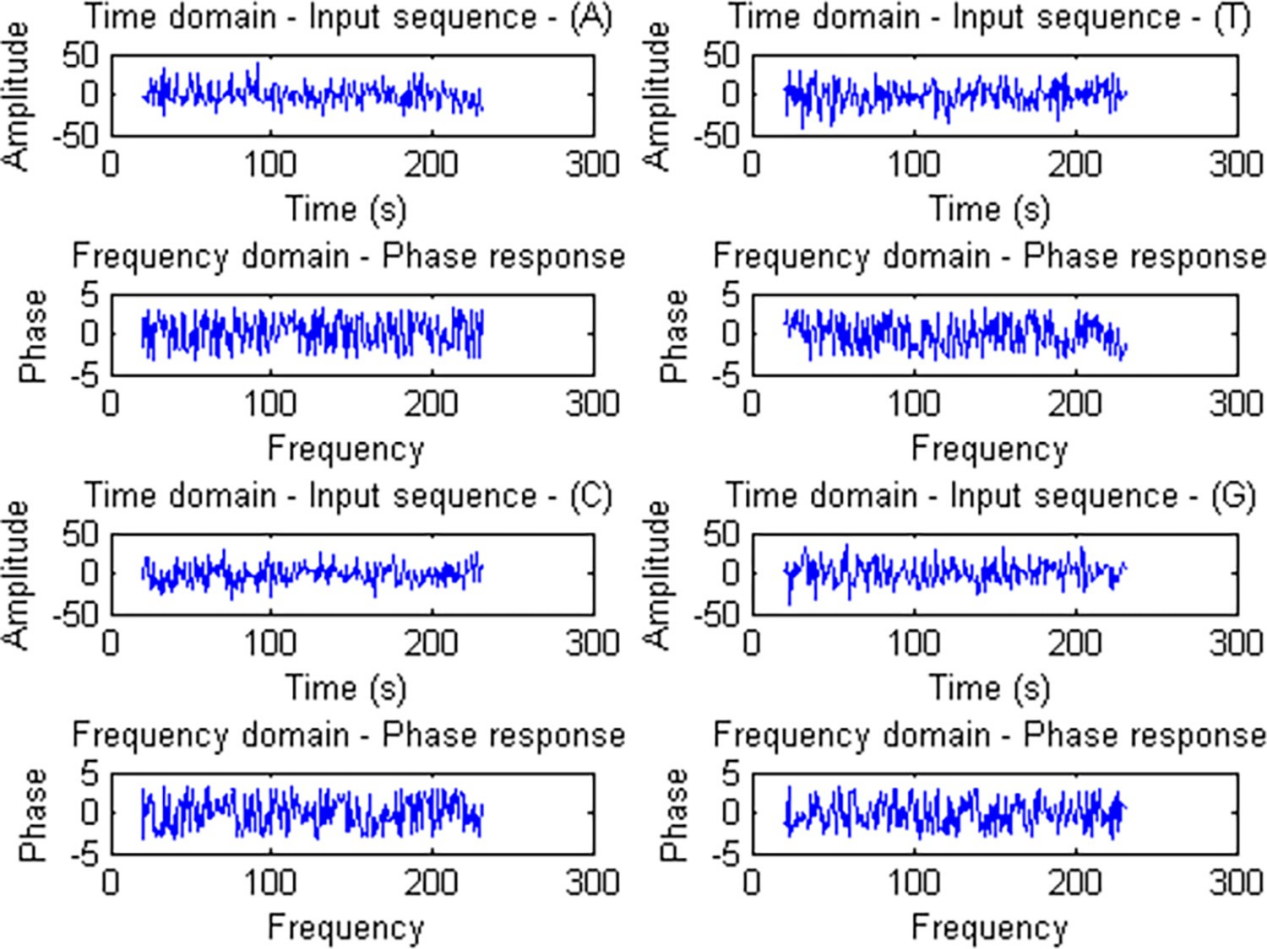
Pair-wise comparison of sequence similarity from Fourier frequency domain for all 30 genes.

**Fig 3:**
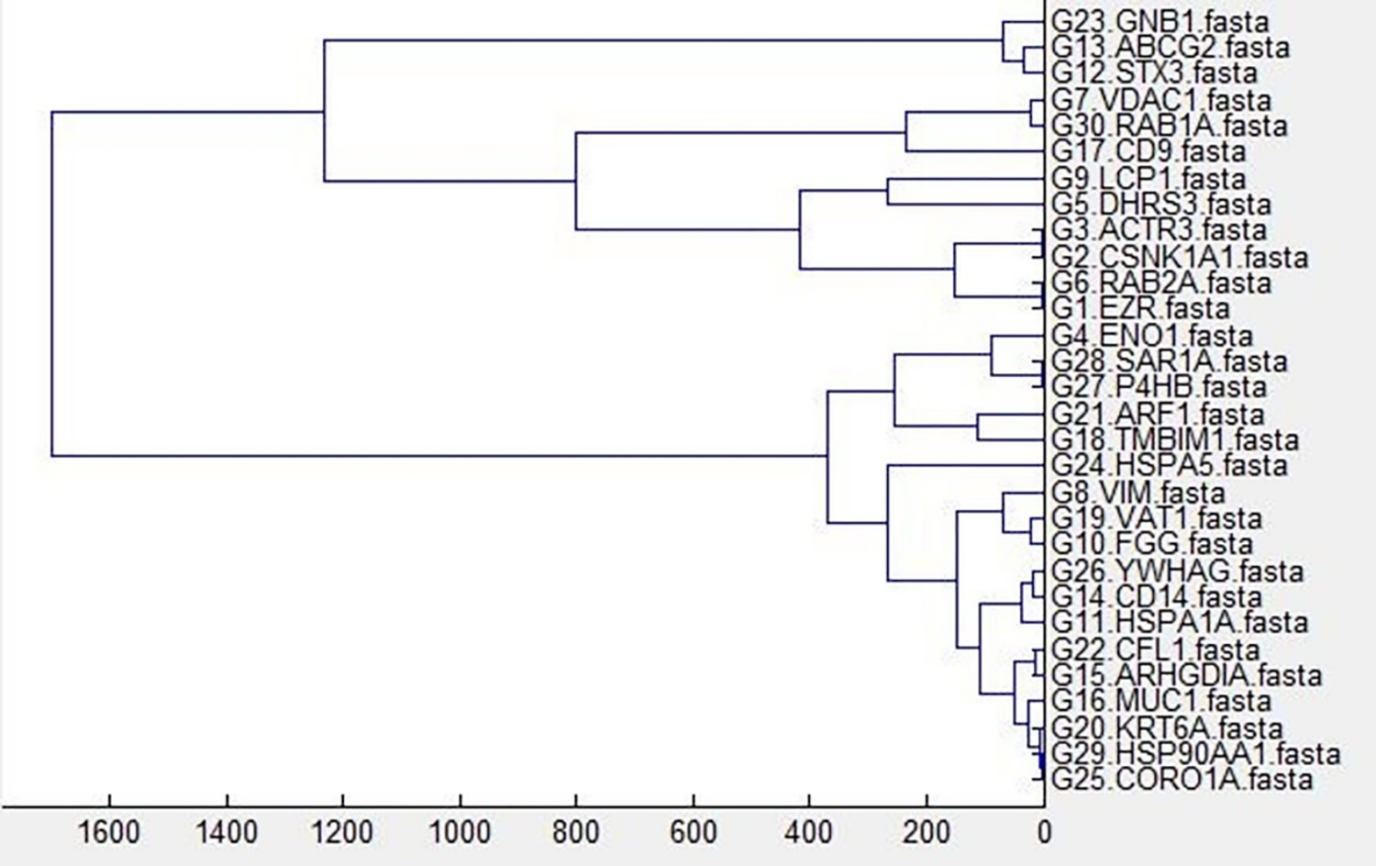
Average clustering based on discrete Fourier transform of bovine candidate gene DNA sequences.

**Fig 4:**
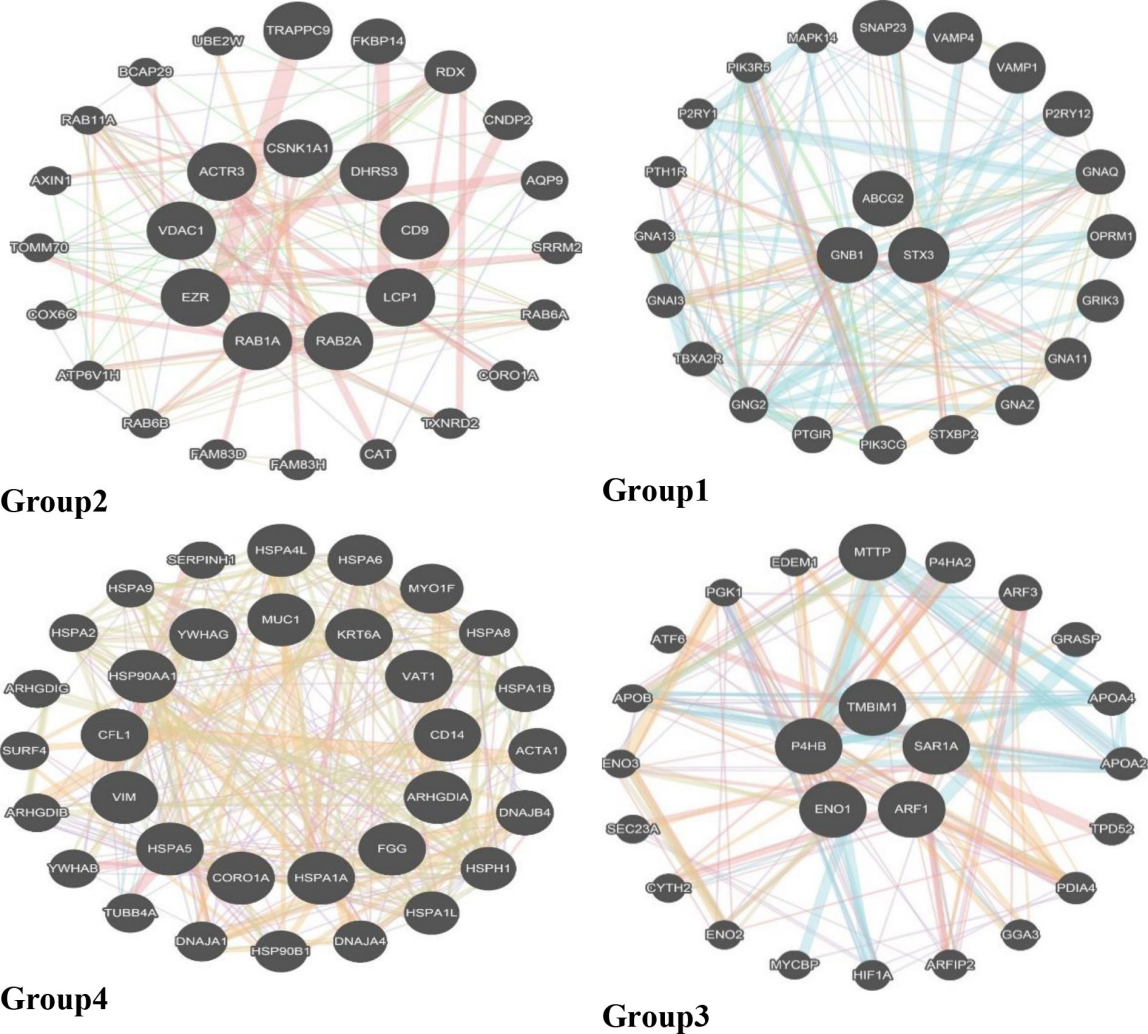
Gene group annotation extraction by GeneMANIA based on DFT based average clustering results.

Generally, Fourier based analysis needs long series data in conjunction with high sampling density. However, this is too much demanding for majority of standard biochemical experiments. In this way, the length of a time series biological experiment cannot be readily extended. However, DNA data, almost lack of these restrictions and working with them are almost simple. Data heterogeneity is intrinsic to the nature of DNA sequence data. Table 1 highlights this issue. Table 1 was created using a package developed within C# to overcome most of the problems caused by data heterogeneity in a way that downstream analysis is readily possible. The Figs 1 and 2 show some general information about the data used in this study in terms of DFT. In this study, *STX3* (79347 bp) and *CD14* (1417 bp) genes were the longest and shortest genes respectively (Table 1). Overall exon number for all investigated genes was 258, with exon 1 of *HSPA1A* (2101 bp) and *HSPA5* (20 bp) genes being the longest and shortest exons respectively. Also, *LCP1* and *ABCG2* genes with 16 exons, and *YWHAG* and *HSPA1A* genes with 1 exon contained the highest and lowest number of exons respectively. Table 2 reflects the four gene groups that were extracted from Fig 3. In this sense, it says that 30 genes were grouped in four gene groups. For each group we used GeneMANIA server to get some clue about their relatedness from biological point of view. Exploration of Fig 4 would indicate that the genes were almost soundly grouped solely based on DNA sequences converted to DFT.

**Table 2:** Gene groups based on DFT average clustering results.

Gene	GROUP1	GROUP2	GROUP3	GROUP4
**1**	STX3	EZR	ENO1	VIM
**2**	ABCG2	CSNK1A1	TMBIM1	FGG
**3**	GNB1	ACTR3	ARF1	HSPA1A
**4**		DHRS3	P4HB	CD14
**5**		RAB2A	SAR1A	ARHGDIA
**6**		VDAC1		MUC1
**7**		LCP1		VAT1
**8**		CD9		KRT6A
**9**		RAB1A		CFL1
**10**				HSPA5
**11**				CORO1A
**12**				YWHAG
**13**				HSP90AA1

**Fig 5:**
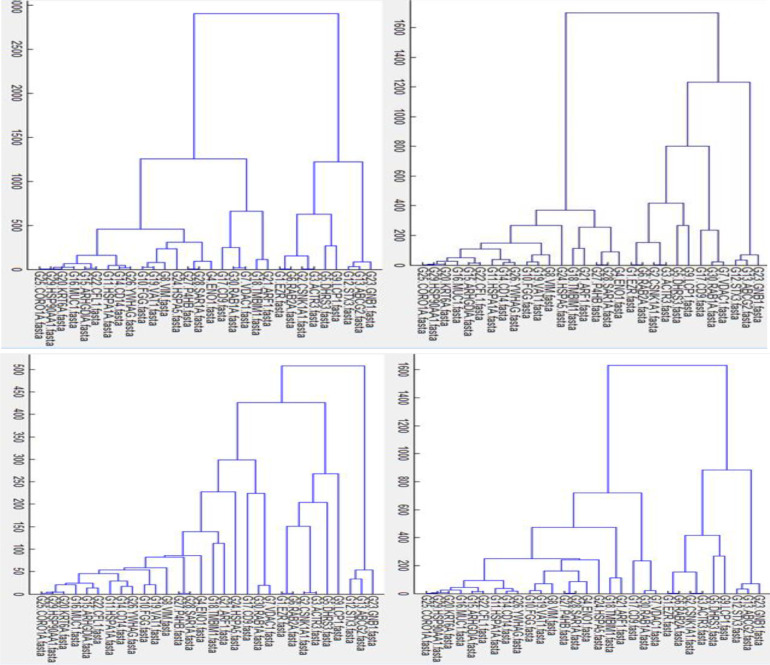
Single, median, complete and Average clustering based on discrete Fourier transform of gene DNA sequences.

Fig 5 show the gene tree results after using Average, Complete, Median and Single algorithms. They show high degree topologies in terms of grouping genes that are tightly clustered over these four algorithms. In this way, the gene tree results due to both Median and Complete algorithms show a high degree of similarity for tightly clustered genes. Visually, it does seem that the clustering algorithms have discovered an obvious pattern of gene grouping. However, in general, we are not expecting hierarchical algorithms to define specific clusters, but rather to define the dendrogram. From any dendrogram we can decipher the proximity or distance between any two groups of genes by looking at the clustering height at which the two groups split. To define clusters, we need to “cut the tree” at some distance and group all samples that are within that distance into groups below. In general, we draw a horizontal line at the height of the cluster that we wish to cut and this defines gene groups. We selected an arbitrary *ad hoc* method for tree cutting that resulted in four gene groups. There is the potential to increase the effective number of gene groups by exploring different ways to select the minimum gene cluster size. It might be better to select the minimum cluster size as a function of the gene dataset size, or perhaps develop a completely separate method for selecting the most appropriate gene group size. In this way, it would be beneficial to make use of a hybrid tree cut [20]. The hybrid tree cut algorithm, similar to dynamic tree cut, creates well shaped clusters with dense cores of tightly lumped nodes and few outliers. It is acknowledged that this algorithm could potentially create clusters in which variables (e.g. genes) are more closely related and thus be more useful for prediction.

**Fig 6:**
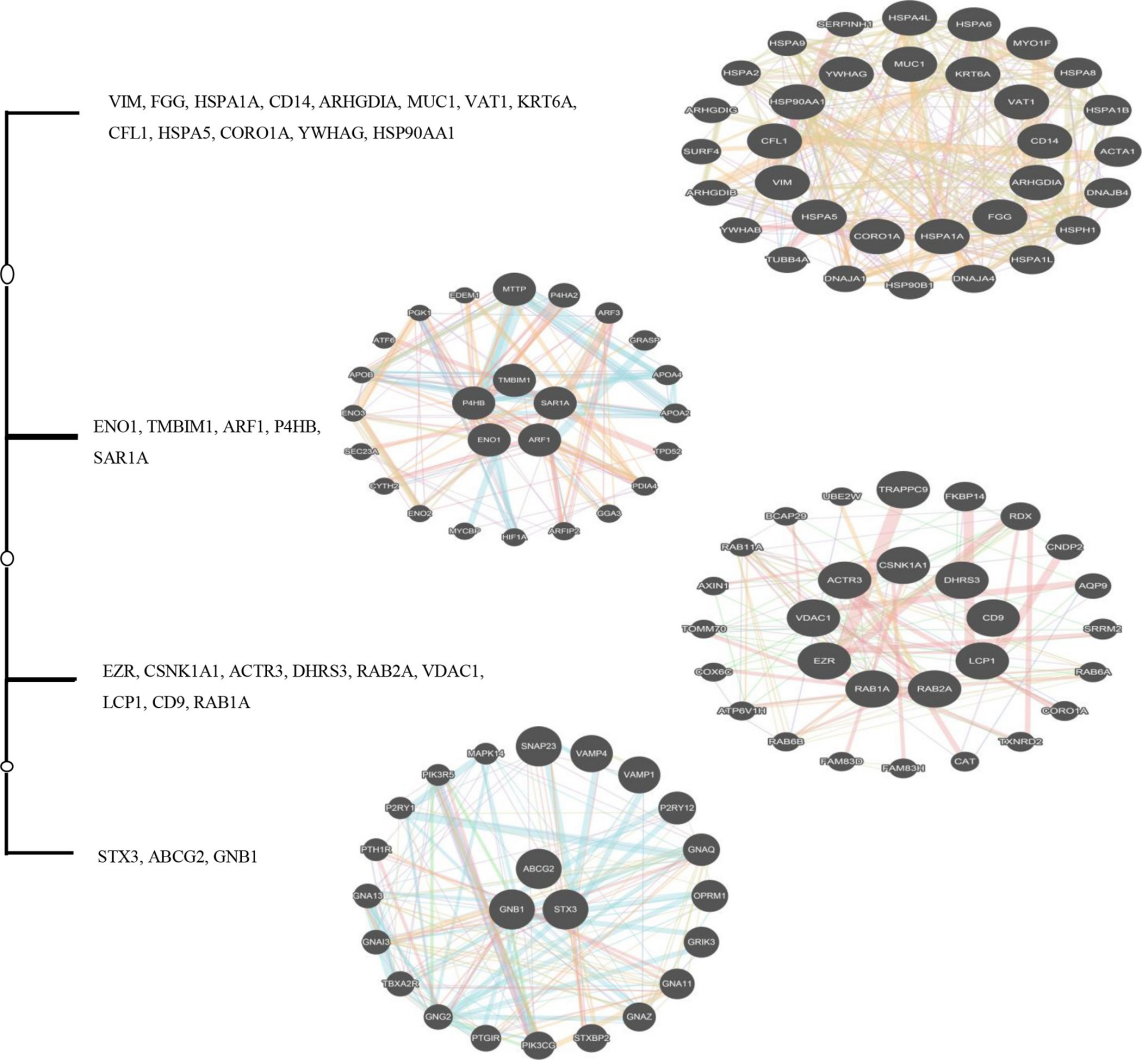
Evidence accumulation integration of different gene clusters.

Gene tree integration by means of evidence accumulation resulted in a gene tree containing four clusters. Fig 5 shows the results. The analyses using GeneMANIA, showed that the gene clusters each shared around 43%, 68%, 52% and 68% co-expression with other genes. All genes within each cluster were associated to the same metabolic pathways. It was also observed that gene clusters functioned differently in terms of being involved in different metabolic pathways. These results could support our gene DNA sequence DFT-based clustering. However, for further explorations of the results, we applied more agglomerative clustering algorithms e.g. complete, median and single to learn the DFT cluster (Fig 5). Fig 6 was created by merging the results from evidence accumulation.

**Fig 7:**
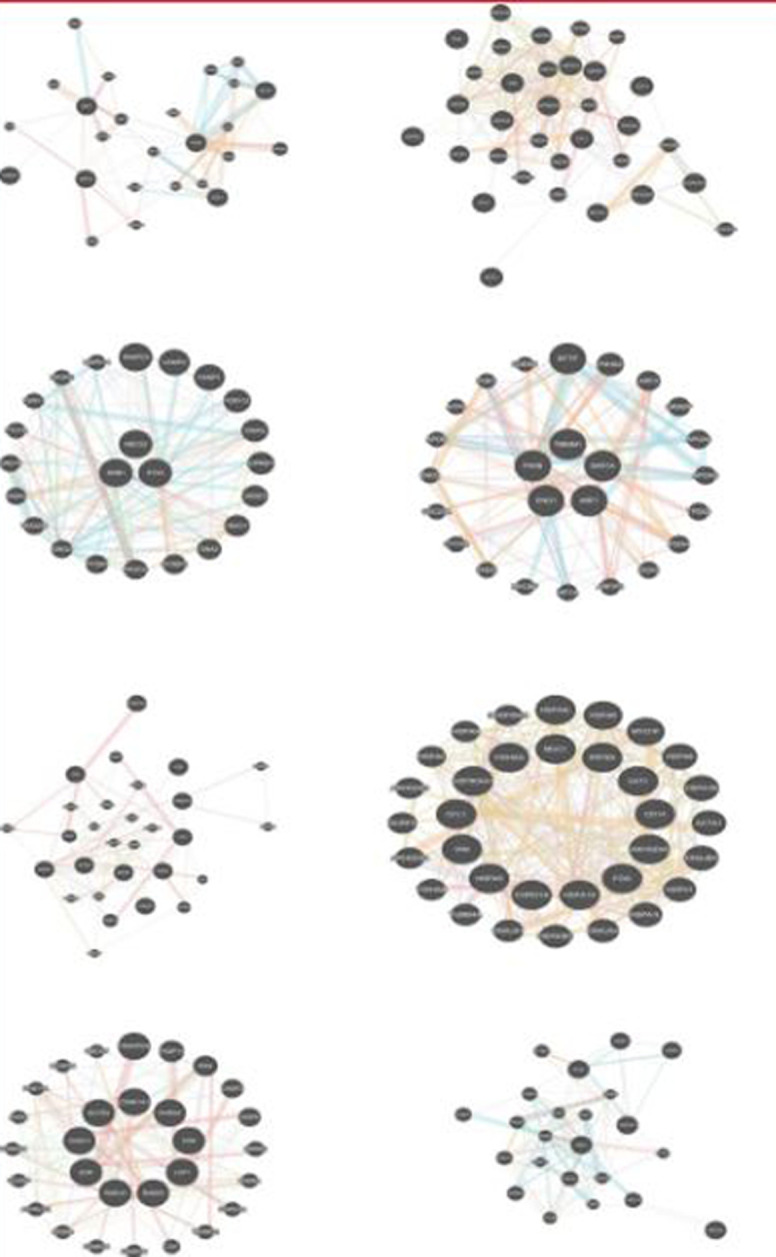
GeneMANIA analysis of evidence accumulation results.

Fig 7 and Table 3 show the final result of combining the four clustering algorithms. For this set of genes, we also tried, in another study, to come up with a gene tree using information theory formalism. In this way, for each gene, the Shannon entropy was calculated in orders one to four miming the Markov chain up to order three orders and, for each gene, the relative Shannon entropy or Kullback-Leibler divergence was calculated. After obtaining the Kullback-Leibler distance for the genes, the results were entered into seven clustering algorithms: single, complete, average, weighted, centroid, median, and k-means. Based on metabolic pathway annotations we saw that the proposed clustering method yielded logical and fast results. This method also doesn’t have the disadvantages of aligning allowed the genes with actual length and content to be considered and also didn’t require high compute memory for long sequences [21]. Different orders of entropies were used to derive information-based gene clustering. These authors used the same *in silico* method of results confirmation as we did in this study [22]. *In silico* confirmation of biological function has found its way in gene association studies, nucleotide polymorphism detection, differential gene expression analysis and novel gene prediction [23–25]. However, there are some discrepancies between the Shannon entropy results and current results, though, with both entropy-based and DFT-based methods, we got logical results only from gene sequence data clustering. The mathematical formalism of Fourier transform has recently been used in biology, mostly in gene expression clustering [14, 17, 26] rather than with individual gene sequences [17]. Lack of thorough biological confirmation, even at the *in silico* level, would remain a big caveat of these explorations. In this study *STX3*, *ABCG2* and *GNB1* cluster together (Fig 6). If we look at the biological knowledge relating to each individual gene, we can see their different molecular functions (https://www.genecards.org/). *STX3* is a member of the syntaxin family and is involved in Sertoli-Sertoli cell junction dynamics and synaptic vesicle cycle pathways. *ABCG2* is included in the superfamily of ATP-binding cassette (ABC) transporters. *GNB1* encodes a beta subunit of guanine nucleotide binding proteins and is an important regulator of the alpha subunits that constitute these proteins. It is associated with signal transduction receptors and effectors and alternative splicing. We believe that by jointly clustering time course gene expression data with their gene DNA sequences will elucidate how these genes function in a way that explains why they are seen to cluster together. Therefore, there are some limitation to both DNA based or time course gene expression individual clustering. For example, fluctuations or changes in gene expression may not necessarily be an indicator of changes in protein levels, since there are so many modifiers involved e.g. post-transcriptional regulation (microRNA mediated regulation). We propose that combing the gene entropy based method and time course gene expression data with the gene DFT method could be an interesting proposal for future research. The simple DFT method applied here can be run easily with the Fast Fourier Transform Algorithm (FFT). FFT is the workhorse of the DFT method. This algorithm can be trained to work readily on these combined levels of data.

**Table 3:** Exploration of gene functions within the four evidence accumulation clusters.

Function of forth cluster	Function of third cluster	Function of second cluster	Function of first cluster
G-protein coupled receptor signaling pathway, coupled to cyclic nucleotide second messenger	GTPase activity	glycolysis	response to unfolded protein
platelet activation	actin filament	glucose catabolic process	response to topologically incorrect protein
heterotrimeric G-protein complex	Golgi vesicle transport	hexose catabolic process	blood microparticle
adenylate cyclase-modulating G-protein coupled receptor signaling pathway	actin filament binding	monosaccharide catabolic process	protein folding
G-protein beta/gamma-subunit complex binding	establishment of endothelial barrier	lipoprotein metabolic process	COP9 signalosome
extrinsic component of cytoplasmic side of plasma membrane	focal adhesion	single-organism carbohydrate catabolic process	unfolded protein binding
extrinsic component of plasma membrane	cell-substrate junction	carbohydrate catabolic process	endocytic vesicle lumen
adenylate cyclase-activating G-protein coupled receptor signaling pathwayextrinsic	cell cortex	cholesterol transporter activity	cytoplasmic membrane-bounded vesicle lumen
component of membrane	endothelial cell development	sterol transporter activity	vesicle lumen
dopamine receptor signaling pathway	cell-substrate adherens junction	lipid transporter activity	establishment of protein localization to mitochondrion
SNARE complex		gluconeogenesis	positive regulation of organelle organization
phospholipase C-activating G-protein coupled receptor signaling pathway		hexose biosynthetic process	mitochondrion organization
specific granule		plasma lipoprotein particle assembly	endoplasmic reticulum-Golgi intermediate compartment
G-protein coupled receptor binding		monosaccharide biosynthetic process	phagocytosis
SNARE binding		protein-lipid complex assembly	
positive regulation of MAPK cascade		very-low-density lipoprotein particle	
GTPase activity		triglyceride-rich lipoprotein particle	
glutamate receptor signaling pathway		endoplasmic reticulum lumen	
lysosomal membrane		glucose metabolic process	
cellular response to fatty acid		plasma lipoprotein particle	
exocytosis		protein-lipid complex remodeling	
response to prostaglandin E		macromolecular complex remodeling	
phosphatidylinositol 3-kinase complex		plasma lipoprotein particle remodeling	
response to prostaglandin		protein-lipid complex	
cellular response to catecholamine stimulus		hexose metabolic process	
positive regulation of cAMP-mediated signaling		monosaccharide metabolic process	
response to catecholamine		plasma lipoprotein particle organization	
regulation of cAMP biosynthetic process		protein-lipid complex subunit organization	
cellular response to monoamine stimulus		carbohydrate biosynthetic process	
vacuolar membrane		regulation of plasma lipoprotein particle levels	
mast cell degranulation		protein folding	
mast cell activation involved in immune response		cholesterol homeostasis	
response to monoamine		sterol homeostasis	
membrane fusion		alcohol binding	
cAMP biosynthetic process		sterol transport	
regulation of cyclic nucleotide biosynthetic process		cholesterol transport	
regulation of cAMP metabolic process		low-density lipoprotein particle remodeling	
cellular response to ketone		triglyceride-rich lipoprotein particle remodeling	
regulation of nucleotide biosynthetic process		very-low-density lipoprotein particle remodeling	
regulation of purine nucleotide biosynthetic process		diterpenoid metabolic process	
mast cell activation		sterol esterification	
regulation of cAMP-mediated signaling		lipid homeostasis	
negative regulation of adenylate cyclase activity		cholesterol esterification	
response to fatty acid		protein heterodimerization activity	
cyclic purine nucleotide metabolic process		steroid esterification	
negative regulation of cyclase activity		retinoid metabolic	
cAMP metabolic process		high-density lipoprotein particle remodeling	
regulation of cyclic nucleotide metabolic process		glycerophospholipid metabolic process	
cyclic nucleotide biosynthetic process		positive regulation of lipid catabolic process	
negative regulation of lyase activity		quaternary ammonium group binding	
vacuolar part		reverse cholesterol transport	
		lipoprotein particle receptor binding	
		positive regulation of steroid metabolic process	
		isoprenoid metabolic process	
		high-density lipoprotein particle	
		detection of visible light	
		cellular response to oxidative stress	
		phospholipid metabolic process	
		phototransduction	
		organic hydroxy compound transport	
		detection of light stimulus	
		phototransduction, visible light	
		process terpenoid metabolic process	

## Conclusions

To best of our knowledge, we are the first to use a DFT-based algorithm to group bovine genes. One possibility worth exploring which would surely influence the effectiveness of the proposed algorithm is the selection of distance measure. As a matter of fact, the distance matrix is very important in the creation of a dendrogram and thus the resulting gene clusters. Selecting the best possible biologically-motivated distance matrix has an important impact on the results. In this study we used Euclidian distance matrix to build up the DFT-based gene trees. A future study would be to investigate the accuracy of the DFT-based gene clustering algorithm with different distance measures. The gene cut size is important. There were some minor discrepancies in the performance of the learned gene tree results, however, we would expect this when the number of genes increases. Gene tree results will most likely be inconsistent over many large gene datasets. This might imply that there is no unique optimal minimum gene cluster size for different large datasets. This matter would certainly be worth investigating to test the validity of this notion, and to determine a way of selecting this optimal minimum cluster gene size.

## Supporting information

S1 File (RAR)
